# Primary mediastinal germ cell tumours: real world experience in the low middle income (LMIC) setting

**DOI:** 10.3332/ecancer.2021.1186

**Published:** 2021-02-11

**Authors:** Anjana Joel, Namrata Mathew, Shalom Sylvester Andugala, Sherin Daniel, Birla Roy Gnanamuthu, Ajoy Oommen John, Josh Thomas Georgy, Raju Titus Chacko, Aparna Irodi, Bijesh Yadav, Subhashini John, Ashish Singh

**Affiliations:** 1 Department of Medical Oncology, Christian Medical College and Hospital Vellore, Vellore 632004, India; 2 Department of Thoracic Surgery, Christian Medical College and Hospital Vellore, Vellore 632004, India; 3 Department of Pathology, Christian Medical College and Hospital Vellore, Vellore 632004, India; 4 Department of Radiology, Christian Medical College and Hospital Vellore, Vellore 632004, India; 5 Department of Biostatistics, Christian Medical College and Hospital Vellore, Vellore 632004, India; 6 Department of Radiotherapy, Christian Medical College and Hospital Vellore, Vellore 632004, India

**Keywords:** Primary mediastinal germ cell tumour, mediastinal seminoma, primary mediastinal nonseminomatous germ cell tumour, low middle income countries

## Abstract

**Purpose:**

Primary mediastinal germ cell tumours (PMGCTs) are rare; with limited data available about their outcomes and optimal treatment in the low middle income countries setting. We studied the clinical profile of patients with PMGCT treated at our centre in order to estimate their survival outcomes and to identify prognostic factors affecting the same.

**Patients and methods:**

Fifty-seven patients with PMGCTs treated between April 2001 and June 2019 were included. Baseline characteristics, details of first line chemotherapy, response rates, toxicity and surgical outcomes were noted. Progression-free survival (PFS) and overall survival (OS) were estimated using the Kaplan–Meier method.

**Results:**

Among 57 male patients (seminoma = 20 and nonseminomatous = 37), the median follow-up was 10 months (range: 1–120 months). For mediastinal seminoma, 9 (45%) and 11 (55%) patients had good and intermediate risk disease, respectively. Nineteen patients (95%) received BEP (Bleomycin, etoposide and cisplatin) chemotherapy. 94.7% had partial responses and median event-free survival was not reached. All patients were alive and disease free at 2 years. For primary mediastinal nonseminomatous germ cell tumours (PMNSGCTs), all patients were poor risk. Thirty-four (91.8%) received BEP/EP chemotherapy as first line. Responses were PRM+ (partial response with elevated markers) in 7 (20.5%) and PRM− in 12 (35.2%). The incidence of febrile neutropenia was 50% and 55.8% in seminole and PMNSGCT, respectively. The median OS was 9.06 months and median PFS was 4.63 months for PMNSGCT. The proportion of patients alive at 1 year and 2 years were 35% and 24.3%, respectively.

**Conclusion:**

Primary mediastinal seminomas are rarer and have better survival outcomes. Treatment of PMNSGCT is still a challenge and is associated with poorer survival outcomes.

## Background

Primary mediastinal germ cell tumours (PMGCTs) account for <5% of all GCTs [1]. They are classified as ‘intermediate or poor risk’ as per the International Germ Cell Consensus Collaborative Group (IGCCCG) just based on their origin in the mediastinum [2]. Though mediastinal seminomas have excellent cure rates and survival outcomes, primary mediastinal nonseminomatous germ cell tumours (PMNSGCTs) which are histologically and cytogenetically similar to gonadal NSGCTs have a dismal prognosis. This is attributed to their more extensive stage at diagnosis, a poorly understood disease biology, compounded by a lack of targetable mutations and a lack of response to immunotherapy as demonstrated in refractory testicular GCTs [3–5].

Mediastinal seminoma responds to initial chemotherapy (BEP/VIP) and relapses though infrequent are often salvaged with second line chemotherapy. The primary treatment of PMNSGCT is multimodality; traditionally induction chemotherapy (BEP/VIP) followed by surgical excision, after documentation of both a radiological and serological response [5]. In the recurrent/refractory setting, salvage chemotherapy with or without high dose therapy (HDT) is standard of care [5]. In low middle income countries (LMIC) like ours, the management of GCTs is more challenging due to the higher proportion of patients with extra mediastinal and extra thoracic involvement at diagnosis of PMGCTs, higher rates of treatment abandonment and attrition, limited access to surgical expertise and salvage chemotherapy with HDT/autologous stem cell transplantation (ASCT) at relapse [6–9]. We reviewed our institutional experience with PMGCTs with an intent to determine the differences in presentation, treatment course and survival outcomes of these patients in the real world LMIC setting.

## Materials and methods

### Study population

We examined the medical records of consecutive adult patients (age >16 years) with PMGCTs treated at our centre from April 2001 to June 2019.

### Baseline characteristics

We collected the following information: demographics, method of preoperative diagnosis/biopsy report, alpha fetoprotein (AFP), beta-human chorionic gonadotrophin (Beta-HCG) and lactate dehydrogenase (LDH) tumour marker values at diagnosis and radiological information. Image guided biopsy was done for all patients with normal serum AFP (<5.5 IU/mL), and beta-HCG < 200 m-IU/mL, who were suspected to have primary mediastinal seminoma. It was not mandatory for patients with an anterior mediastinal mass with elevated serum AFP or beta-HCG > 200 m-IU/mL, diagnosed to have PMNSCGT, to undergo an image guided biopsy of the mediastinal mass prior to initiation of chemotherapy. All patients underwent contrast enhanced CT imaging of the thorax +/− abdomen/pelvis, prior to and after completion of all chemotherapy. End of treatment fluorodeoxy glucose-positron emission tomography (FDG-PET) CT (EOT PET CT) was done after completion of chemotherapy only in patients with seminoma. All patients were stratified as good, intermediate or poor risk as per the IGCCCG classification based on their serum markers and tumour type. The last date of outpatient clinic visit was taken as the date of last follow up. For patients not returning to hospital, the follow up was by telephonic interview.

### Treatment details

The details of initial chemotherapy regimens, duration of treatment and best response (pre- and post-chemotherapy tumour markers and imaging) were collected. Radiological response assessment was performed using radiological assessment according to the Response Evaluation Criteria in Solid Tumours version 1.1. Overall disease response was documented for patients with PMNSGCT based on the combination of radiological response and tumour markers as: complete response (CR), partial response with normal tumour markers (PRM−), partial response with plateau of tumour markers (PRM+) and progressive disease (PD). Adverse events during treatment were documented and graded using the Common Terminology Criteria for Adverse Events, version 4.02.

The surgical details, tumour stage, surgical pathology (including preoperative biopsy and postresection histology) of patients with PMNSCGT who underwent post chemotherapy surgical resection were collected. Post-operative chemotherapy was administered to patients whose surgical histopathol ogy showed residual viable tumour. Among patients who relapsed or were refractory to first line chemotherapy, the details of salvage chemotherapy and outcomes were collected.

### Survival outcomes

We defined an event as disease relapse or progression or death due to any cause; if the death occurred before disease progression; or the last follow-up date, whichever was earlier. Patients who were lost to follow-up were censored on the date of their last follow-up. Data was censored on 30 April 2020. We calculated event-free survival (EFS) from the date of initiation of treatment to the date of the event and overall survival (OS) was calculated from the date of histopathological diagnosis to the date of death. Patients who were still alive at the end of the study were censored on the date of the last follow up. We included patients who abandoned treatment in our survival analysis; since their inclusion is more reflective of the real world scenario [10].

### Statistical analysis

Data was entered and analysed using SPSS Version 23. Categorical and continuous variables were summarised using descriptive statistics. Disease-free survival and OS were calculated from the time of diagnosis using the Kaplan–Meir method. Time-to-event analysis was done using the Kaplan–Meier estimator, and hazard ratios were calculated using the Cox proportional model.

## Results

**Figure 1.**
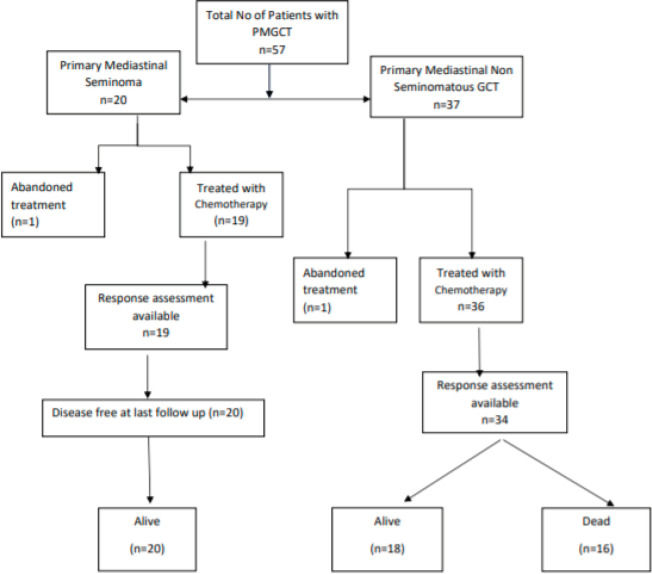
STROBE diagram for study population (STROBE, strengthening the reporting of observational studies in epidemiology).

Fifty-seven patients were diagnosed with mediastinal GCT at our centre from April 2001 to June 2019. Among these, 20 were diagnosed with primary mediastinal seminoma and 37 as PMNSGCT based on elevated tumour markers and pre-operative biopsy (Figure 1).

### Patient characteristics

All the patients were male. The median age at diagnosis was 25 years (13–44) and 22 years (15–49) for seminoma and PMNSGCT, respectively. The commonest presenting symptoms were cough, breathlessness and systemic symptoms in both groups of patients (Table 1). There were distant metastases in 10% and 40% of patients with mediastinal seminoma and PMNSGCT, respectively. None of our patients had associated leukaemia or myelodysplastic syndrome or Klinefelter’s syndrome, indicating the rarity of these associations (Tables 1 and 2).

### Histologic and tumour marker evaluations

Among patients with PMNSGCT, the median serum AFP, B-HCG, LDH at diagnosis were 13,682 IU/mL (range: 0.69–89,500), 60.3 m-IU/mL (range: 0.8–81,940) and 912 U/L (range: 265 to 4,341), respectively. Preoperative biopsy was done in all patients with mediastinal seminoma. The baseline B-HCG was >1,000 m-IU/mL in 6 (16.2%) patients; all with PMNSGCT (Table 1).

### IGCCCG risk stratification

Among patients with mediastinal seminoma, 11 (55%) belonged to ‘intermediate risk’ and 9 (45%) to ‘good risk’ as per IGCCCG risk criteria. All 37 patients with PMNSGCT were stratified as ‘poor risk’ based on IGCCCG criteria (Table 3).

### Chemotherapy details

All patients received cisplatin-based chemotherapy as first line treatment. Nineteen patients with mediastinal seminoma received either three cycles of BEP or four cycles of EP chemotherapy. Among patients with PMNSGCT, majority (91.9%) received either four cycles of BEP or three cycles BEP followed by one cycle EP and two patients received non bleomycin containing first line chemotherapy (VIP/TIP (Paclitaxel, ifosfamide and cisplatin)). Prophylactic pegfilgrastim on Day 6 was used in patients receiving EP chemotherapy. Among patients on BEP chemotherapy, routine pegfilgrastim was not used and filgrastim was used as indicated during episodes of febrile neutropenia or during delayed recovery of white blood cell counts. One patient with seminoma and two with PMNSGCT received one cycle of pre-phase chemotherapy with single agent carboplatin AUC 5 in view of poor Eastern Cooperative Oncology Group (ECOG) performance status; before initiation of standard chemotherapy. The median number of cycles of first line chemotherapy was four in both groups (Table 4).

### Post chemotherapy evaluation and responses

Majority of patients (75%) with seminoma had a partial response as per radiological assessment. Ten patients with seminoma underwent EOT PET CT; all of whom were interpreted as PET positive. These patients, however, have remained on surveillance with subsequent CT imaging showing further decrease in size of the residual mass and are all currently in remission. Among PMNSGCT, response assessment was based on both radiology and tumour markers. There was one patient with a radiological CR with normal tumour markers. Partial radiological response with normal tumour markers (PRM−) and with plateau of tumour marker decline (PRM+) was seen in 12 (35.2%) and 7 (20.5%) patients, respectively. Eleven (29.7%) had primary refractory disease. Among the patients with PRM+ (*n* = 7), three had tumours which were more necrotic and cystic on CT imaging. Among 12 patients with PRM−, six had tumours which were more necrotic and cystic on CT imaging.

### Post chemotherapy management

Fourteen patients with seminoma remained on surveillance. Two patients were given radiotherapy to the residual mediastinal mass and four patients underwent surgery; all their surgical biopsies showed extensive fibrosis with necrosis. Following the introduction of the SEMPET trial, EOT-PET CT was done in 10 patients; at a median time of 10 weeks (range: 8–16 weeks) from the date of last chemotherapy. All these patients were interpreted as EOT PET positive based on the standardised uptake value uptake (as per the SEMPET definition) but remained on surveillance; subsequent imaging has shown further decrease in size of the residual mediastinal mass. Post chemotherapy surgery was done in 12 (32.4%) patients with PMNSGCT (Table 5). Surgical resection was R0, R1 and R2 in 33%, 16% and 25%, respectively. There was viable tumour in two patients (16%) and in three patients the surgery was done at another centre and the histopathology was not available. The remaining seven patients had necrosis or mature teratoma. Six patients (17.6%) did not undergo surgery despite being in remission. Two patients had minimal low volume residual disease and chose to remain on follow up, two had residual multiple cystic liver lesions and bilateral lung metastases, respectively, and two refused surgery.

### Toxicity

Febrile neutropenia rates were similar across both seminoma and PMNSGCT groups. The incidence of all grades of anaemia and thrombocytopenia, including Grade 3 and 4 was higher in the PMNSGCT subset (Table 3). The incidence of Grade 3/Grade 4 neutropenia was 45% (9 out of 20 patients) and 54% (20 out of 37 patients) in seminoma and PMNSGCT, respectively. There were no deaths attributed to febrile neutropenia in either group. The incidence of Grade 3 acute kidney injury was 38.8% and 20.5% among seminoma and PMNSGCT subgroups, respectively. None of these patients required haemodialysis.

### Salvage therapy

Sixteen patients with PMNSGCT received salvage chemo for refractory disease or disease progression/relapse. Two patients received TIP chemotherapy, one received Gem-ox chemotherapy and 11 patients received Vinblastin, ifosfamide and cisplatin chemotherapy. The median number of cycles was 3. Five out of these 16 patients underwent surgery after salvage chemotherapy. There was viable tumour on histopathology in one patient, no tumour in three patients and one patient had surgery elsewhere. None underwent HDT and ASCT.

### Survival outcomes

The median follow up for the entire patient cohort was 10.54 months (range: 1–178 months).

Seminoma: There was no EFS events in this cohort. The median EFS was not reached (range: 1–177 months) and all patients were alive at 2 years.

**Figure 2.**
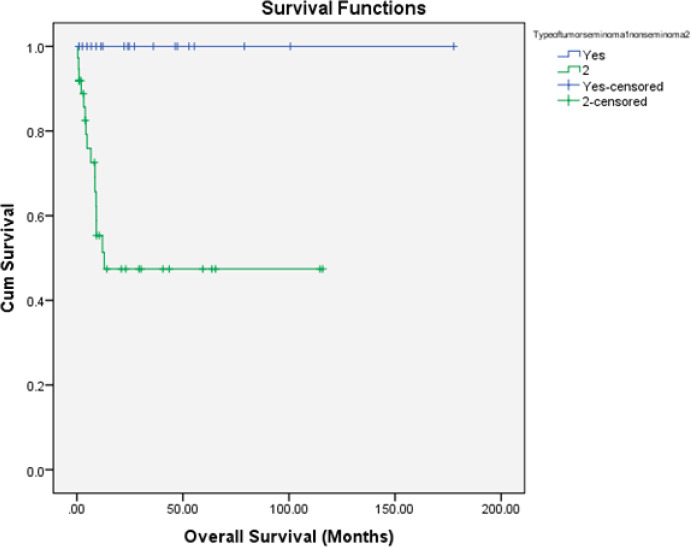
Kaplan–Meier OS estimate according to histology (blue line: seminoma and green line: PMNSGCT).

**Figure 3.**
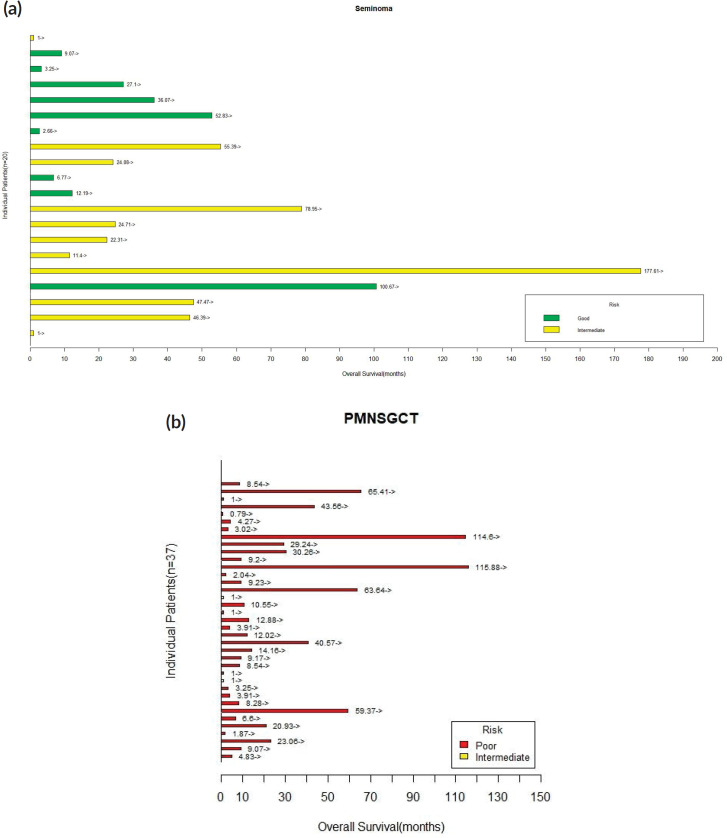
(a): Swimmer’s plot depicting OS for patients with mediastinal seminoma. (b): Swimmer’s plot depicting OS for patients with PMNSGCT.

Nonseminomatous tumours: There were 19 (51.4%) progression-free survival (PFS) events and 16 deaths (43.2%) among the entire cohort of 37 patients. The median OS was 9.06 months and median PFS was 4.63 months. The proportion of patients alive at 1 year and 2 years were 35% and 24.3%, respectively. The details are shown in Figures 2 and 3.

## Discussion

Primary GCTs of the mediastinum are associated with a poor prognosis when compared to gonadal GCTs [2, 5]. Even in developed nations, the multidisciplinary management of these tumours poses a challenge. Though GCTs among the adolescent and young population are treated with a curative intent worldwide, the additional challenges faced in LMIC settings are unique and include delayed presentation, higher proportion of treatment abandonment, poor follow up and limited access to more advanced salvage treatments like HDT and ASCT (HDT/SCT) [6–8, 11]. Our study reports real world outcomes among patients with PMGCTs in a LMIC setting.

Mediastinal seminoma is associated with a good prognosis with infrequent relapses [12]. Our patients with mediastinal seminoma (*n* = 20) had a larger median tumour size at diagnosis and higher proportion of patients with ‘intermediate risk’ disease as per the IGCCCG stratification. All our patients with mediastinal seminoma were treated with BEP/EP chemotherapy and consistent with prior literature, had the best outcomes with a median EFS of 24.39 months with no relapses during the follow up period. The incidence of false positivity on EOT PET CT was high and active surveillance is an acceptable option for these patients [13].

Our patients with PMNSGCT (*n* = 37) had more advanced disease at diagnosis as reflected by higher mean AFP/HCG, higher incidence of extra mediastinal disease and distant metastases. All were stratified as ‘poor risk’ based on the IGCCCG risk stratification. These factors are well recognised as predictors of poor survival outcomes and disease recurrence [14]. We used BEP induction chemotherapy for four cycles for majority of our patients and saw mostly partial responses. We noted that even though tumour markers plateaued at the end of first line chemotherapy with minimal radiological regression, these tumours became more cystic and necrotic.

The role of surgery following chemotherapy is crucial to direct subsequent therapeutic decisions [15]. In addition to quantifying response to induction chemo, surgery also helps debulk chemo resistant tumour; especially when this is limited to the mediastinum. Single centre experiences reported from Indiana University and Memorial Sloan Kettering Cancer Center best illustrate the role of post chemotherapy surgery towards improving survival outcomes and cure rates in PMNSGCT including a subset of chemo refractory patients with localised intra-thoracic disease, if their mediastinal primary is resectable [15–18]. Approximately a third of our patients with PMNSGCT underwent surgical resection. There was residual viable tumour in 16.6% of our patients; in contrast to the higher rates described (30%–54.8%) [15–20].

We observed that the incidence of haematological toxicity (i.e.) anaemia (all grades and G3/4) and thrombocytopenia (all grades and G3/4) were high. The rates of febrile neutropenia were high in both seminoma and PMNSGCT in comparison to Western data. Compared to poor risk patients with gonadal primary GCTs and lung metastases, the need and extent of pulmonary surgery among patients with extra gonadal NSGCTs is much more extensive and may hence predispose these patients to a greater risk of drug induced pneumonitis [15]. There is a debate on whether omitting bleomycin among these patients who are planned for a major thoracic surgery consisting of thoracotomy followed by surgical excision of these tumours, reduces perioperative pulmonary complications [15, 21]. There was no severe bleomycin related pulmonary toxicity among our patients; this could be an underestimation since DLCOs (diffusing capacity for carbon monoxide) were not routinely monitored before and after chemotherapy.

The survival outcomes among patients with PMNSGCT worldwide have not changed dramatically even in the post cisplatin era [9, 14–18, 22, 23]. Despite improvements in early diagnosis, availability of cisplatin-based chemotherapy, advancements in surgical expertise and SCT, the 5 year OS has not crossed the 60% mark [14–18, 22, 23]. It is also evident that patients undergoing surgical resection have a better outcome when compared to those who did not undergo surgery [17, 18].

In the Asian subcontinent, Liu *et al* [22] have also shown that though the 5-year survival among patients with primary mediastinal seminoma is 87%, the 5 year survival among patients with nonseminomatous mediastinal GCTs was only 27%. However, our outcomes among PMNSGCT in the real world LMIC setting are worse; with median OS 9.06 months and median PFS 4.63 months. The proportion of our patients alive at 1 year and 2 years is also inferior to those described *a priori* [9].

Even though 16 patients received salvage/second line chemotherapy, none received HDT/ASCT; which has been shown to be acceptable as a second line strategy and has produced long standing remissions [24–26]. The lack of targetable mutations and lack of efficacy of immunotherapy among patients with relapsed/refractory GCTs coupled with the higher proportion of patients presenting with adverse prognostic factors at diagnosis including extra thoracic disease, indicate the importance of an intensive first line treatment approach inclusive of surgical resection, if a curative intent of treatment is to be attempted [4, 5]. Also, patients who required salvage chemotherapy prior to surgery have an inferior PFS when compared to those who did not require salvage chemotherapy prior to surgery [17, 18].

In LMIC settings like ours, the management of GCTs is more challenging, given the higher rates of treatment abandonment as seen among our patients with testicular cancer in India; despite their curative intent of treatment [6]. This is attributed to the predominance of younger patients, some of whom have to support a dependent family and the need to often travel long distances for their treatment. We acknowledge that our study is limited by its retrospective nature, small sample size, short follow up durations and limited interpretation of toxicity data. However, for rare tumours like PMGCTs, generation of real-world data plays a significant role in further refining our therapeutic approach toward the multimodality management of these patients and also highlights the need for collaboration among high volume centres; especially in LMIC settings; in order to improve our outcomes.

## Conclusion

In our study, patients with PMNSGCT had a poorer outcome in comparison to mediastinal seminoma. This was predominantly due to a higher proportion of patients presenting with extra-thoracic disease and inability to achieve a complete surgical resection among patients with extra mediastinal disease. Outcomes in mediastinal seminoma are favourable; with chemotherapy alone producing excellent cure rates.

## Conflicts of interest

All authors have no conflicts of interest to declare.

## Funding

The authors have not received any financing to carry out this work.

## Authors’ contributions

Anjana Joel: conceptualization, methodology, formal analysis, investigation, writing - original draft, visualization.

Namrata Mathew: conceptualization, methodology, formal analysis, investigation, writing - review & editing, visualization, supervision, project administration, funding acquisition.

Shalom Sylvester Andugala: conceptualization, methodology, formal analysis, writing - review & editing, visualization.

Sherin Daniel: conceptualization, methodology, formal analysis, investigation, writing - review & editing, visualization, supervision.

Birla Roy Gnanamuthu: conceptualization, methodology, formal analysis, investigation, writing - review & editing, visualization.

Ajoy Oommen John: conceptualization, methodology, investigation, writing - review & editing, visualization.

Josh Thomas Georgy: conceptualization, methodology, investigation, writing - review & editing, visualization.

Raju Titus Chacko: methodology, formal analysis, investigation, writing - review & editing, supervision, visualization.

Aparna Irodi: methodology, formal analysis, investigation, writing - review & editing, visualization.

Bijesh Yadav: formal analysis, data curation, software.

Subhashini John: methodology, formal analysis, investigation, writing - review & editing, visualization.

Ashish Singh: writing - review & editing, supervision, project administration, funding acquisition, resources.

## Figures and Tables

**Table 1.** Baseline characteristics (*N* = 57).

	Seminoma (*n* = 20)	Nonseminoma (*n* = 37)
Median age in years (range)	25 (13–44)	22 (15–49)
Gender, Male	20(100%)	37 (100%)
Patients who underwent pretreatment biopsy	20 (100%)	31 (83.8%)
Yolk sac histology	NA	11 (29.7%)
Smokers	4 (20%)	5 (13.5%)
Other risk factors	Crypto-orchidism seen in two individuals	Autism seen in one individual
Presenting symptoms (%) • Cough • Breathlessness • Systemic symptoms (fever/LOW/LOA) • Chest pain • Voice change • Others	11 (55%) 10 (50%) 5 (25%) 11 (55%) 2 (10%) 7 (35%)	20 (54.1%) 12 (34.1%) 13 (35.1%) 18 (45.6%) 2 (5.4%) 9 (24.3%)
ECOG at diagnosis (%)		
• 1	18 (90%)	21 (56.8%)
• 2	1 (5%)	10 (27%)
• 3	1 (5%)	3 (8.1%)
• 4	0	3 (8.1%)
Median haemoglobin (gm%) (range)	13.05 (10.67–16.5)	11.4 (7.6–16.1)
Median AFP (median, range)	1.67 (0.8–9.59)	13,682 (0.69–89,500)
Median HCG (median, range)	32.1 (0.2–466)	60.3 (0.8–81,940)
Median LDH (median, range)	1,366 (288–4,733)	912 (265–4,341)

LOW: loss of weight; LOA: loss of appetite

**Table 2.** Disease extent (*n* = 57).

	Seminoma (*n* = 20)	Nonseminoma (*n* = 37)
Median tumour size (in cm) (range)	Median 12.5 (7–23)	Median 12 (5–20)
Nodes involved (%)		
• Mediastinal	2 (10)	7 (18.9)
• Nonmediastinal	7 (35)	5 (13.5)
• None	11 (55)	25 (67.6%)
SVCO present (%)	8 (40)	9 (24.3%)
Other local complications (%)		
• Pericardial	6 (30)	11 (29.7%)
• Pleural or lung	5 (25)	17 (45.9%)
• Vocal cord palsies	2 (10)	1 (2.7%)
• Others (atrial, rib, spinal cord)	1 (5)	9 (24.3%)
• None	9 (45)	7 (18.9)
• Two or more of the above	3 (15)	6 (16%)
Distant metastases present, outside mediastinum (%)	2 (10%)	15 (40%)
Sites of distant metastases (osseous, visceral, both, none)		
• Osseous	1	2 (5.4%)
• Visceral^a^	1	8 (21.6%)
• None	0	5 (13.5%)
• Both osseous and visceral	0	22 (59.5%)
Brain metastases present	0	3 (8.1%)
HCG ≥ 1,000	x	5 (13.5%)
IGCCCG risk staging (%)		
• Good risk	9 (45%)	0
• Intermediate risk	11 (55%)	0
• Poor risk	X	37 (100%)

SVCO: superior vena cava obstruction

a One patient had a 3mm indeterminate lung nodule.

**Table 3.** First line chemotherapy, responses and toxicity.

	Seminoma (*n* = 20)	Nonseminoma (*n* = 37)
Prephase	1 (5%)	2 (5.4%)
Median number of cycles of first line chemotherapy	3.95 (0 to 6)	4 (1to 6)
Abandoned treatment	1 (5%)	1 (2.7%)
First line chemotherapy regimen • EP • VIP • BEP	6 0 13	13 2 21
Tumour marker trend at completion of first line chemotherapy	*N* = 19	*N* = 34
• Normal	16 (84.2%)	10 (27%)
• Plateau	0	19 (51.3%)
• High	2 (10.5%)^a^	2 (5.8%)
• Not done	1 (8.3%)	3 (8.1%)
Radiological response after first line chemotherapy	*N* = 19	*N* = 34
• CR	1 (5.3%)	1 (2.7%)
• PR	18 (94.7%)	17 (45.9)
• SD	0	2 (5.4%)
• PD	0	11 (29.7%)
• Not done	0	6 (16.2%)
Overall disease response (imaging + markers) after first line chemotherapy	*N* = 19	*N* = 34
• CR	1 (5.3%)	1 (2.9%)
• PRM−	16 (84.2%)	12 (35.2%)
• PRM+	2 (10.5%)	7 (20.5%)
• PD	0	10 (29.4%)
• Not done^b^	0	4 (12%)
Haematologic toxicities		
• Febrile neutropenia	9 (50%)	19(55.8%)
• Neutropenia (G3 or G4)	9 (45%)	20 (54%)
• Anaemia (any grade)	8 (44.4%)	26 (76.4%)
• Anaemia (G3 or G4)	2 (11.1%)	22 (64.7%)
• Thrombocytopenia (any grade)	9 (50%)	20 (58.8%)
• Thrombocytopenia (any grade/G3 or G4)	4 (22.2%)	16 (47%)
Non haematologic toxicities		
• Pneumonitis	1 (5.5%)	2 (5.8%)
• Peripheral neuropathy	0	3 (8.8%)
• Acute kidney injury/renal failure Grade 3	7 (38.8%)	7 (20.5%)

a Persistent elevated LDH in seminoma.

b Patients did not undergo reassessment

**Table 4.** Post first line chemotherapy course in mediastinal seminoma.

	Seminoma
Surveillance	14 (90%)
Radiation therapy	2 (10%)
Surgical resection	4
EOT PET done	10
Median time from last chemo to PET CT	12 weeks (8–16 weeks)
EOT PET CT interpretation as ‘positive’	10

**Table 5.** Post first line chemotherapy course in PMNSGCT.

	Nonseminoma (*n* = 34)
Surveillance	6 (17.6%)
Died before reassessment	2 (5.8%)
Surgical resection	12 (32.4%)
• R0	4
• R1	2
• R2	3
• No information^a^	3
Surgical histopathology (*n* = 12) • No residual tumour • Residual tumour • Teratoma • Not known	5 (41.6%) 2 (16.6%) 2 (16.6%) 3 (25.2%)
Salvage chemotherapy	16

a Surgery done at another centre
